# The microglial component of amyotrophic lateral sclerosis

**DOI:** 10.1093/brain/awaa309

**Published:** 2020-12-02

**Authors:** Benjamin E Clarke, Rickie Patani

**Affiliations:** 1 Department of Neuromuscular disease, Institute of Neurology, University College London, Queen Square, London, UK; 2 The Francis Crick Institute, 1 Midland Road, London, UK

**Keywords:** microglia, ALS, astrocyte, ageing

## Abstract

Microglia are the primary immune cells of the CNS, carrying out key homeostatic roles and undergoing context-dependent and temporally regulated changes in response to injury and neurodegenerative diseases. Microglia have been implicated in playing a role in amyotrophic lateral sclerosis (ALS), a neurodegenerative disease characterized by extensive motor neuron loss leading to paralysis and premature death. However, as the pathomechansims of ALS are increasingly recognized to involve a multitude of different cell types, it has been difficult to delineate the specific contribution of microglia to disease. Here, we review the literature of microglial involvement in ALS and discuss the evidence for the neurotoxic and neuroprotective pathways that have been attributed to microglia in this disease. We also discuss accumulating evidence for spatiotemporal regulation of microglial activation in this context. A deeper understanding of the role of microglia in the ‘cellular phase’ of ALS is crucial in the development of mechanistically rationalized therapies.

## Teaser

Clarke and Patani review the involvement of microglia in ALS, focusing specifically on evidence for spatiotemporally regulated neurotoxic and neuroprotective roles. Understanding how microglia contribute to the ‘cellular phase’ of ALS promises to advance the development of therapies with a mechanistic rationale.

## Introduction

Amyotrophic lateral sclerosis (ALS) is a rapidly progressing neurodegenerative disease involving the degeneration of both upper and lower motor neurons in the motor cortex, brainstem and spinal cord (Al-Chalabi *et al.*, 2017). Loss of motor neurons results in extensive paralysis commencing usually focally in the limbs or bulbar muscles. ALS is a universally fatal disease, typically due to respiratory failure between 2 and 5 years after diagnosis (Niedermeyer *et al.*, 2019). Most cases of ALS (>90%) are sporadic, but study of familial forms of the disease has been critical in identifying causative mutations, now numbering more than 30 genes. Mutations in *C9orf72*, *SOD1*, *TARDBP* and *FUS* are the most common genetic forms, accounting for more than half of familial ALS cases. Although motor neurons are primarily affected in ALS, non-neuronal cells play a key role in disease progression (Philips and Rothstein, 2014; Beers and Appel, 2019). Motor neuron death itself, and the pathogenic cascade within surviving motor neurons, are accompanied by an inflammatory response. Evidence for this from blood and CSF of ALS patients is well established (Tateishi *et al.*, 2010; Gupta *et al.*, 2011; Lu *et al.*, 2016). This includes the activation of CNS resident microglia and astrocytes. Here, we will focus primarily on the microglial contribution to ALS, before discussing cellular interplay with motor neurons, astrocytes, oligodendrocytes and peripheral immune cells invading the CNS. It is clear that neurodegeneration can trigger inflammation and that inflammation itself can contribute to the process of neurodegeneration. The issue of whether inflammation might cause neurodegeneration in ALS remains unresolved. The finding of ALS-related gene mutations being expressed in microglia is consistent with a possible upstream role of neurodegeneration, but more work is required here to gain a more comprehensive insight (Cady *et al.*, 2014).

Microglia are the primary immune cells of the CNS with critical roles in the protection of neurons from infection or injury and in synaptic regulation (Davalos *et al.*, 2005; Nimmerjahn *et al.*, 2005; Schafer *et al.*, 2013). Unlike other resident cells of the CNS, microglia do not originate from the ectoderm and instead arise from primitive macrophages in the mesodermal yolk sac, invading the CNS during development (Ginhoux *et al.*, 2010). Microglial identity is thought to manifest through both an ontological basis and refinement by local CNS cues (Bennett *et al.*, 2018). Significant brain regional heterogeneity of mouse (Nikodemova *et al.*, 2014; Grabert *et al.*, 2016) and human (Böttcher *et al.*, 2019) microglia has been reported, with recent single cell studies suggesting that this diversity is far higher in younger mice and in ageing/disease states than during normal adulthood (Ajami *et al.*, 2018; Hammond *et al.*, 2019; Li *et al.*, 2019). Microglia are highly motile cells that constantly survey their micro-environment by continuously extending and retracting their processes, phagocytosing dead cells and debris. In response to a multitude of stimuli, microglia can become activated, undergoing graded and temporal changes in their morphology and gene expression in response to injury, infection or neurodegenerative diseases (Szalay *et al.*, 2016; Mathys *et al.*, 2017; Ajami *et al.*, 2018; Hammond *et al.*, 2019; Haruwaka *et al.*, 2019). Microglia can also become proliferative when activated and release a wide variety of cytokines that have subsequent protective or detrimental effects on neurons (Li and Barres, 2018). The exact response of microglia to certain stimuli is context-dependent and may be determined by several factors including the chronicity of the stimulus, ageing and regional heterogeneity. Here, we review the literature specifically concerning the contribution of microglia to the pathological process of ALS.

## Evidence from human ALS tissue

Study of post-mortem tissue has provided an association between microglia and ALS. However, there has been some controversy over whether microglial activation from a ramified or stellate to an ameboid shape, increased proliferation and/or upregulation of inflammatory pathways, is present in post-mortem ALS tissue. While there have been some reports of microglial proliferation only occurring in a subset of ALS cases (Spiller *et al.*, 2018; Tam *et al.*, 2019), other studies have reported that microglia are activated in both the motor cortex (Dols-Icardo *et al.*, 2020) and spinal cord (Brettschneider *et al.*, 2012; D'Erchia *et al.*, 2017) of sporadic ALS cases. Controversy over increases in microglial activation in ALS may be due to heterogeneity across different ALS genetic subtypes or perhaps due to artefacts in immunolabelling or degradation of post-mortem tissue. Furthermore, since microglia have been shown to undergo large changes in gene expression in normal brain ageing (Soreq *et al.*, 2017), different ages of patients may affect the conclusions made from these studies.

Findings from post-mortem tissue are limited as they only provide a snapshot of pathology at the end stage of disease and are also, to some degree, affected by variable agonal phases and post-mortem intervals. These limitations have been overcome to some extent by monitoring neuroinflammation in living patients. To this end, advances in PET imaging have provided some information about longitudinal changes in neuroinflammation in ALS patients. Increased neuroinflammation has been observed in the primary and supplementary motor and temporal cortices of ALS patients using PET imaging of various ligands that bind to the immune marker, translocator protein 18 (TSPO) (Turner *et al.*, 2004; Corcia *et al.*, 2012; Alshikho *et al.*, 2018). However, TSPO labels astrocytes and other immune cells in addition to microglia, making it impossible to delineate microglial specific effects. In summary, the study of ALS patients has so far only provided limited information concerning the specific involvement of microglia in ALS.

## Lessons from animal models of ALS

Animal models have provided invaluable insights into microglial involvement in ALS. Mouse and human microglia are thought to share a large majority of genes, suggesting that animal models are a useful tool for the study of microglia (Galatro *et al.*, 2017). However, it is important to note that differences in genes involved in immune function were found to exist between mouse and human microglia, which may be particularly relevant to neurodegenerative diseases such as ALS. Furthermore, much of what is known about the mechanisms of microglial involvement in ALS comes from studies of mouse models overexpressing mutant ALS proteins (Table 1). Although this caveat is partially addressed by using cells overexpressing wild-type protein as a control, the study of microglia expressing mutant genes at physiological levels is lacking.

**Table 1:** Summary of studies investigating microglia using animal models of ALS

Gene/model	Microglial phenotype	Reference
***SOD1***		
SOD1^G93A^	Increased MAC1/CD11b immunoreactivity in grey and white matter of the spinal cord at symptomatic ages.	Almer *et al.*, 1999; Alexianu *et al.*, 2001
	Increased proliferation of CD11b^+^ CD45^lo^ cells.	Chiu *et al.*, 2008
	CSFR1 inhibitor, GW2580, reduced microglial proliferation, partially rescued motor neuron death and slowed disease progression.	Martínez-Muriana *et al.*, 2016
	Elimination of part of the proliferating CD11b population had no effect on motor neuron degeneration.	Gowing *et al.*, 2008
	Wild-type bone marrow transplant into mSOD1/PU1 knockout mice partially ameliorated motor neuron loss and extended disease duration.	Beers *et al.*, 2006
	Spatial transcriptomics reveal changes in microglia before motor neurons in the ventral spinal cord.	Maniatis *et al.*, 2019
	Shift in high and low expressing Iba1^+^ cells preceding disease onset.	Gerber *et al.*, 2012
	Microglia of mSOD1 mice secrete more ROS and NO than wild-type microglia and kill co-cultured motor neurons.	Beers *et al.*, 2006; Xiao *et al.*, 2007
	Adult but not neonatal primary microglia kill co-cultured motor neurons via a NF-κB dependent mechanism.	Frakes *et al.*, 2014
SOD1^G93A^, SOD1^A4V^	Extracellular aggregated mSOD1 increases TNF-α production in primary microglia.	Roberts *et al.*, 2013
SOD1^G93A^, SOD1^G85R^	Extracellular mSOD1 alters primary microglial morphology, stimulates superoxide and cytokine release and induces neurotoxicity.	Zhao *et al.*, 2010
SOD1^G37R^	Removal of mSOD1 from CD11b expressing cells increased late stage disease duration.	Boillee *et al.*, 2006
***TARDBP***		
TDP-43^WT^, TDP-43^M337V^, TDP-43^A315T^	Both wild-type and mutant TDP-43 stimulate cytokine production in primary microglia.	Zhao *et al.*, 2015
TDP-43^WT^, TDP-43^G348C^, TDP-43^A315T^	Mice overexpressing wild-type and mutant TDP-43 display increased Iba-1 immunoreactivity at symptomatic ages.	Swarup *et al.*, 2011*a*
TDP-43^−/−^	Microglia-specific knockout of *TARDBP* enhances amyloid clearance in an Alzheimer’s disease mouse model.	Paolicelli *et al.*, 2017
rNLS8	Microglia proliferate in the recovery phase of a reversible neuronal specific TDP-43ΔNLS overexpression mouse model. Blocking microgliosis was detrimental suggesting that microglia were neuroprotective.	Spiller *et al.*, 2018
***FUS***		
FUS^WT^	Astrocyte conditioned media from wild-type FUS treated cells exacerbates proinflammatory induction of primary microglia.	Ajmone-Cat *et al.*, 2019
	Mice overexpressing FUS display increased CD68 immunoreactivity in the spinal cord.	Mitchell *et al.*, 2013
^Δ^FUS^(1-359)^	RNA sequencing of mice overexpressing truncated FUS suggests neuroprotective presymptomatic and proinflammatory symptomatic stages in microglia.	Funikov *et al.*, 2018
***C9orf72***		
*C9orf72* ^−/−^	C9orf72 knockout mice display a robust immune phenotype including enlarged microglial lysosomes and increased expression of proinflammatory cytokines.	O'Rourke *et al.*, 2016
GA‐CFP	Iba-1 immunoreactivity and altered morphology occurred in mice expressing poly-GA, which was prevented by vaccination.	Zhou *et al.*, 2020
C9^450C^*C9orf72*^−/−^	Increased Iba1 immunoreactivity of mice expressing C9orf72 repeat expansions and lack of endogenous C9orf72.	Zhu *et al.*, 2020

Most work on microglia in ALS has been performed in the SOD1G93A mouse, with rodent models of TARDBP, FUS and C9orf72 developed more recently. ROS = reactive oxygen species.

### SOD1

Mutations in *SOD1* were the first discovered to cause ALS (Rosen, 1993). Mouse models expressing mutant SOD1 (mSOD1) recapitulate key features of the disease including motor neuron loss, paralysis and premature death (Gurney *et al.*, 1994). Elegant studies have concluded that neuron-specific expression of mSOD1 is not sufficient to recapitulate the full disease observed in mSOD1 mice and that non-neuronal cells play an important part in mSOD1 disease progression (Pramatarova *et al.*, 2001; Clement *et al.*, 2003; Yamanaka *et al.*, 2008).

Microglial activation has been observed from the onset of disease in mSOD1 mouse models (Almer *et al.*, 1999; Alexianu *et al.*, 2001; Chiu *et al.*, 2008). Removal of mSOD1 from CD11b expressing microglia using a Cre-lox system lengthened lifespan, but did not affect the onset of disease, suggesting that microglia are most important in the later phases of disease in mSOD1 mice (Boillee *et al.*, 2006). Furthermore, pharmacological inhibition of CSFR1, a protein with roles in microglial proliferation, survival and maturation, reduced microglial activation and extended survival of mSOD1 mice (Martínez-Muriana *et al.*, 2016). Proliferation of microglia during the symptomatic phase of disease may not play an important role in disease progression as ablation of part of the proliferating microglial cell population using mSOD1/CD11b-TK^mt-30^ mice treated with ganciclovir at symptomatic ages did not affect motor neuron survival (Gowing *et al.*, 2008). However, as chronic systemic administration of ganciclovir was lethal, local delivery intrathecally into the spinal cord by osmotic pump was used instead in this study. This treatment left a significant number of proliferating microglia unaffected. Therefore, a reduced population of proliferating microglia may still be sufficient to effect the same level of damage in mSOD1 mice.

Expression of mSOD1 in microglia alone is not sufficient to cause motor neuron degeneration. Replenishment of microglia by transplantation of mSOD1 bone marrow at birth into PU1knockout microglia-deficient mice was not able to cause motor neuron loss even though transplanted mSOD1 microglia were morphologically more activated, with short ramified processes, than wild-type microglia (Beers *et al.*, 2006). In terms of onset, survival and duration of disease, mSOD1/PU1knockout mice given mSOD1 bone marrow transplantation showed no differences to mSOD1 mice. However, mSOD1/PU1 knockout mice given wild-type bone marrow transplants survived for longer, had an extended disease duration and decreased motor neuron loss compared to mSOD1/PU1knockout mice given mSOD1 bone marrow transplants, suggesting that removal of mSOD1 from microglia is protective (Beers *et al.*, 2006). While disease onset was reported not to be affected, there was a trend towards a delayed onset of symptoms in mSOD1/PU1 knockout mice given wild-type bone marrow transplants. However, it is important to note that this reduction in damage to mice given wild-type bone marrow transplants cannot be attributed solely to microglia as PU1 knockout affects all myeloid and lymphoid cells.

While early studies suggested that microglia only had a role in the late stages of disease, changes in microglia that occur before motor neuron loss may still be significant to disease pathomechanisms. Recent spatial transcriptomics of the mSOD1 mouse spinal cord showed that changes in microglial gene expression preceded changes in motor neurons (Maniatis *et al.*, 2019). Furthermore, microglial numbers have been reported to be decreased before disease onset in mSOD1 mice (Gerber *et al.*, 2012). Therefore, changes in microglia in the early disease phase, while more subtle, may still be relevant in ALS.

Further evidence for early phenotypes of mSOD1 microglia has come from studies of primary cultures from neonatal mSOD1 mice. Neonatal wild-type microglial cultures were toxic to motor neurons in co-culture after exposure to exogenous mSOD1 (Zhao *et al.*, 2010; Roberts *et al.*, 2013). Furthermore, neonatal microglia overexpressing mSOD1 were toxic to motor neurons in co-culture under basal conditions and were more toxic than activated wild-type microglia after stimulation by the bacterial endotoxin, lipopolysaccharide (LPS) (Xiao *et al.*, 2007). However, another study found no differences in motor neuron survival in co-cultures with neonatal mSOD1 microglia, but instead reported neurotoxicity when co-cultured with adult mSOD1 microglia (Frakes *et al.*, 2014). One potential limitation of mSOD1 mouse models is that mutations within the *SOD1* gene, along with mutations in *FUS*, fall within the overwhelming minority of cases that do not exhibit the pathological hallmark of TDP-43 nuclear-to-cytoplasmic mislocalization. It follows that other gene mutations may capture ALS pathomechanisms with greater fidelity and precision.

### TARDBP

Mutations in *TARDBP*, encoding TDP-43, only account for ∼5% of familial ALS cases, but mislocalization of wild-type TDP-43 is observed in >95% of all ALS cases (Neumann *et al.*, 2006). TDP-43 is an RNA-binding protein involved in several roles in RNA regulation. Both wild-type and mutant TDP-43 have been reported to stimulate cytokine production in primary mouse microglia (Zhao *et al.*, 2015). Furthermore, mice overexpressing wild-type or mutant TDP-43 displayed increased immunoreactivity of microglia at symptomatic ages (Swarup *et al.*, 2011*a*). It has been suggested that TDP-43 plays an important role in microglial phagocytosis, since microglial-specific knockout of *TARDBP* enhanced amyloid clearance and synaptic loss in an Alzheimer’s disease mouse model (Paolicelli *et al.*, 2017). In a reversible mouse model of doxycycline suppressible neuronal human TDP-43 containing a defective nuclear localization signal, only small changes in microglial activation were observed preceding and during an aggressive disease course of 50% motor neuron loss and death within 10 weeks (Spiller *et al.*, 2018). However, when TDP-43 expression was switched off during disease, mice recovered motor function and microglia became activated and cleared neuronal TDP-43. Microglia were shown to directly play a neuroprotective role in this process as pharmacological suppression of microglia negatively affected recovery of motor function (Spiller *et al.*, 2018).

### FUS

Mutations in another RNA binding protein, *FUS*, also account for close to 5% of familial ALS cases (Kwiatkowski *et al.*, 2009; Vance *et al.*, 2009). Interestingly, mislocalization of FUS has been recently found to occur in motor neurons of sporadic ALS cases, suggesting a role in the majority of ALS cases (Tyzack *et al.*, 2019). However, the importance of microglial FUS in ALS is only beginning to be understood. Exacerbation of proinflammatory cytokine production occurred in primary microglial cultures after incubation with astrocyte conditioned media from astrocyte cultures overexpressing wild-type FUS (Ajmone-Cat *et al.*, 2019). Mice overexpressing wild-type FUS also display microglial activation in the spinal cord (Mitchell *et al.*, 2013). Furthermore, both pro- and anti-inflammatory microglial activation has been reported in mice expressing a truncated FUS protein lacking a nuclear localization signal, both before and after disease onset (Funikov *et al.*, 2018), suggesting that mislocalization of FUS is sufficient to result in microglial activation.

### C9orf72

An intronic hexanucleotide repeat expansion in *C9orf72* is the most common cause of familial ALS cases (DeJesus-Hernandez *et al.*, 2011; Renton *et al.*, 2011). It is thought that both a gain-of-function mechanism through production of dipeptide repeats (DPRs) and a loss-of-function mechanism contribute to C9orf72-mediated pathology (Balendra and Isaacs, 2018). In mice, C9orf72 is highly expressed in microglia and while no neuronal death is observed in *C9orf72^−/−^* mice, microglial activation has been reported (O'Rourke *et al.*, 2016). In a mutant C9orf72 mouse model expressing the neurotoxic DPR, poly-GA, immunization against poly-GA reduced microglial activation (Zhou *et al.*, 2020). Furthermore, in a recent study, reduction of C9orf72 expression in a mouse model with a repeat expansion resulted in hippocampal microglial activation, implicating both gain and loss of C9orf72 function in modulating microglial activation (Zhu *et al.*, 2020).

## Microglial interactions with other cell types in ALS

### Astrocytes

**Figure 1:**
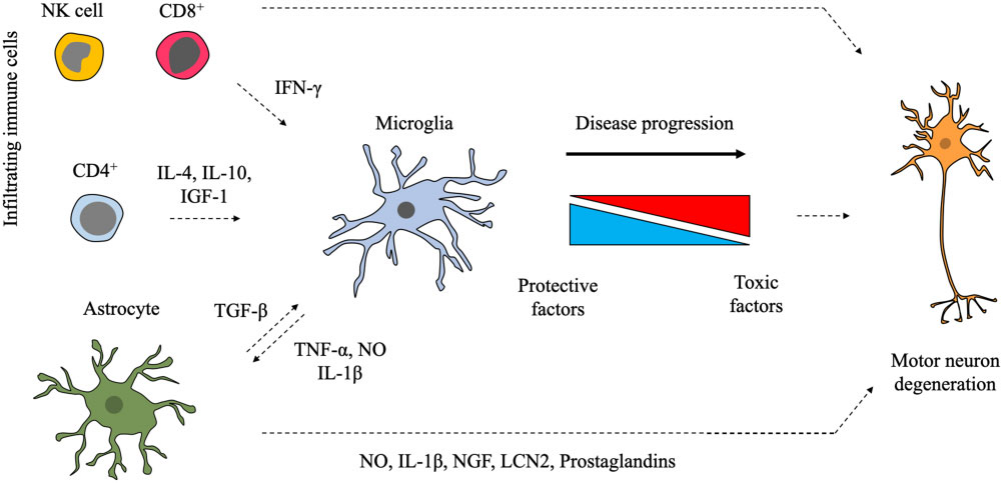
**Multiple cell types affect microglial responses in ALS.** Both astrocytes and peripheral immune cells that infiltrate the CNS during ALS disease progression can affect microglial responses, resulting in an overall protective or toxic microglial phenotype. Changes in cellular composition, ageing and factors released from dying cells are also likely to affect the microglial phenotype in ALS. Components of this figure were created using Servier Medical Art templates, which are licensed under a Creative Commons Attribution 3.0 Unported License.

Astrocytes are the most abundant glial cell in the CNS, providing multiple mechanisms of support to neurons including metabolic homeostasis, synapse regulation and immune functions (Vasile *et al.*, 2017). In an elegant study, it was demonstrated that microglia are able to influence astrocytic gene expression, resulting in both the loss of homeostatic functions and a transformation to a neurotoxic state (Liddelow *et al.*, 2017). Like microglia, astrocyte dysfunction has been implicated in non-cell autonomous motor neuron death in ALS (Nagai *et al.*, 2007; Yamanaka *et al.*, 2008). Both loss of homeostatic functions and a toxic release of factors have been implicated in astrocytic non-cell autonomous mechanisms of motor neuron death in ALS (Serio and Patani, 2018). As significant crosstalk is known to occur between microglia and astrocytes (Jha *et al.*, 2019), microglia-astrocyte crosstalk is likely to play a role in ALS (Fig. 1).

The interaction between microglia and astrocytes has been partially addressed in the mSOD1 mouse model. While no differences were observed in the morphology of astrocytes following removal of mSOD1 from microglia (Boillee *et al.*, 2006), reducing microglial proliferation in the mSOD1 mouse decreased activation of astrocytes (Gowing *et al.*, 2008). Concomitantly, removal of mSOD1 from astrocytes resulted in a decrease in microglial activation (Yamanaka *et al.*, 2008). Further evidence for the influence of astrocytes on microglial activation comes from studies where transplantation of wild-type astrocyte precursors into the mSOD1 mouse spinal cord reduced microglial activation (Lepore *et al.*, 2008) and transplantation of mSOD1 expressing astrocyte precursors into wild-type mice was sufficient to induce microglial activation (Papadeas *et al.*, 2011).

The mechanisms that dictate the interplay between astrocytes and microglia in ALS are not well understood. While microglia release multiple factors that are able to influence astrocytic gene expression (Jha *et al.*, 2019), none have been demonstrated to directly manipulate astrocytic gene expression in ALS models. Conversely, astrocytic release of TGF-β has been shown to affect microglia as an astrocyte-specific overexpressing TGF-β mSOD1 mouse reduced the expression of a subset of microglial factors that have been proposed to be neuroprotective (Endo *et al.*, 2015). Somewhat surprisingly, owing to the vastly different embryological origin, microglia have been reported to undergo a fate transition to astrocytes when cultured from symptomatic mSOD1 rats (Trias *et al.*, 2013). In aggregate, these studies argue for a deeper understanding of the cellular interplay and primacy of events between microglia and astrocytes.

### Oligodendrocytes

Oligodendrocytes are responsible for providing metabolic support to neurons and maintaining the myelin sheath required for neuronal saltatory conduction of action potentials. Degeneration of oligodendrocytes has been found to occur in mSOD1 mice (Kang *et al.*, 2013). This degeneration was accompanied by microglial activation and microglial localization to apoptotic oligodendrocytes. Furthermore, in this study removal of mSOD1 specifically from oligodendrocytes resulted in a delay in disease onset, which coincided with delayed microglial activation. Proliferation of oligodendrocyte progenitor cells (OPCs) was found to occur in the mSOD1 model to replace lost oligodendrocytes (Kang *et al.*, 2013). Microglial reactive state can influence OPC differentiation as directing microglia to pro- or anti-inflammatory states respectively inhibited or promoted remyelination in the lysolecithin model of demyelination (Miron *et al.*, 2013; Lombardi *et al.*, 2019). However, the relationship between microglia and OPCs in the context of ALS is not well understood.

### Peripheral immune cells

Breakdown of the blood–brain barrier during ALS disease progression enables the infiltration of peripheral immune cells into the CNS (Kawamata *et al.*, 1992; Engelhardt *et al.*, 1993; Garofalo *et al.*, 2020). Invading immune cells have been implicated in both neurotoxic and neuroprotective responses in ALS (Beers *et al.*, 2008, 2011*a*; Coque *et al.*, 2019), which is more comprehensively reviewed elsewhere (Thonhoff *et al.*, 2018; Beers and Appel, 2019). Interestingly, peripheral macrophages are reported to become ‘microglia-like’ and express several previously described microglial-specific genes following their entry into the CNS during chronic neuroinflammation in the experimental autoimmune encephalomyelitis (EAE) model of multiple sclerosis, which may also be relevant in diseases such as ALS (Grassivaro *et al.*, 2020).

Several studies have suggested that peripheral immune cells affect microglial morphology and gene expression during mSOD1 disease progression. Increased expression of microglial factors associated with neurotoxicity was observed at end stage in mSOD1/PU1knockout mice given bone marrow transplants from either *Rag2*^−/−^ or *Cd4*^−/−^ mice (Beers *et al.*, 2008), which are unable to generate mature lymphocytes or CD4^+^ T cells, respectively. This suggests that these cells reduce harmful microglial activation in the mSOD1 mouse model. Interestingly, while mSOD1 mice lacking T cells increased factors associated with neurotoxicity there was a reduction in morphological activation, suggesting a disconnect between morphological change and activation at the level of gene expression in this context.

The regulation of microglia by T cells in the mSOD1 mouse may be temporally mediated, as mSOD1/RAG2 knockout mice had a delayed disease onset, which coincided with an increase in microglial activation associated with a neuroprotective state (Tada *et al.*, 2011). The reason for this discrepancy in microglial activation state between early and late disease in mSOD1 mice may be a temporal shift in the composition of the infiltrating immune cell population during disease progression. It is important to note that these results are controversial as the finding of delayed disease onset in mSOD1/RAG2 knockout mice is in direct contradiction to findings of earlier disease onset in other studies using mSOD1/RAG2 knockout mice (Beers *et al.*, 2008; Chiu *et al.*, 2008), which was suggested to be due differences in environmental factors or the copy numbers of the SOD1 transgene between the strains of mice used (Tada *et al.*, 2011).

It has been suggested that protective CD4^+^ cells enter the CNS at an earlier stage of disease progression than cytotoxic CD8^+^ cells, which results a shift in microglial activation from a neuroprotective to a neurotoxic state (Beers *et al.*, 2008; Liao *et al.*, 2012). A recent study has suggested that NK cells also shift microglial responses towards a neurotoxic state in mSOD1 mice, in addition to directly causing neurotoxicity through a perforin-dependent release of lysosomes (Garofalo *et al.*, 2020). Depletion of the NK cell population with blocking antibodies reduced microglial proliferation, altered morphology and shifted microglial gene expression towards a phenotype associated with neuroprotection, coinciding with extended survival of mSOD1 mice. Together, these studies show that infiltrating immune cells affect microglial phenotypes, which correlate with modifying disease in mSOD1 models of ALS.

### Motor neurons

As previously stated, motor neurons are the primary cell type affected in ALS. Interestingly, while transcriptional changes in astrocytes and oligodendrocytes occurred after changes in motor neurons in mSOD1 mice (Sun *et al.*, 2015), recent spatial transcriptomics has suggested that microglial dysfunction precedes changes in motor neurons (Maniatis *et al.*, 2019). However, it is unlikely that microglia are the dominating cell type directly driving motor neuron death in ALS. As previously mentioned, expression of mSOD1 in microglia alone does not result in disease (Beers *et al.*, 2006) and there is a lack of microglial-mediated motor neuron death in *C9orf72^−/−^* mice, even though widespread microglial activation occurs (O'Rourke *et al.*, 2016). Furthermore, systemic neuroinflammation involving microglial activation is also observed in other diseases such as lupus and rheumatoid arthritis while no motor neuron death occurs (Schwartz *et al.*, 2019).

Nevertheless, microglia may still play an important modulatory role in motor neuron death in ALS. The expression of mSOD1 specifically in motor neurons is not sufficient to cause disease and non-neuronal cell types including microglia have been shown to affect disease progression in mSOD1 mice (Pramatarova *et al.*, 2001; Clement *et al.*, 2003; Boillee *et al.*, 2006). Thus, pathological mechanisms in both motor neurons and surrounding glia are required for disease to occur. This is supported by the high expression of ALS-associated genes such as *C9orf72*, *OPTN* and *TBK1* in both motor neurons and glia. The molecular mechanisms dictating microglial-mediated protection or toxicity to motor neurons in ALS are discussed below.

## Microglial molecular changes in ALS

Changes in microglial gene expression in response to different stimuli have been described as M1 or M2 states, corresponding to whether they are deleterious or protective to neurons, respectively. However, this categorization of microglial states is regarded as an oversimplification as it is clear that microglia elicit graded and context-dependent responses when activated (Ransohoff, 2016; Ajami *et al.*, 2018). It is more helpful to regard this as a reactive continuum, of which M1 and M2 are polar phenotypes. Nevertheless, specific gene expression signatures are likely to exist in ALS, across different subtypes of the disease and simultaneously involving both detrimental and protective factors (Chiu *et al.*, 2013). The microglial expression profile has been shown to change during disease progression, as microglia from mSOD1 mice initially express an overall protective phenotype but undergo a transformation to an overall toxic state (Liao *et al.*, 2012). This temporal transformation of microglial activation states has also been observed in other models of neurodegenerative conditions using single cell analysis, including models of Alzheimer’s disease (Mathys *et al.*, 2017), the EAE model of multiple sclerosis and a model of Huntington’s disease (Ajami *et al.*, 2018), with heterogeneity in activation states likely to be due to the chronicity and type of stimulus. Furthermore, microglia may elicit region-specific differences in response to either inflammatory cues or in disease states (Beers *et al.*, 2011*b*; Nikodemova *et al.*, 2014; Clarke *et al.*, 2019). To add further complexity, certain factors may be protective or toxic in a stimulus-dependent manner. For example, the phosphoglycoprotein osteopontin has been shown to be detrimental in the EAE model but is protective in a model of spinal cord injury (Chabas *et al.*, 2001; Hashimoto *et al.*, 2007).

### Factors primarily associated with microglial protection in ALS

Several factors including cytokines, secreted proteins, receptors and transcription factors are altered in ALS post-mortem tissue and mSOD1 models. It is important to note that the expression of these factors is not restricted to microglia and it is likely that many secreted proteins and cytokines released from astrocytes or infiltrating immune cells also play an important role in disease.

**Figure 2:**
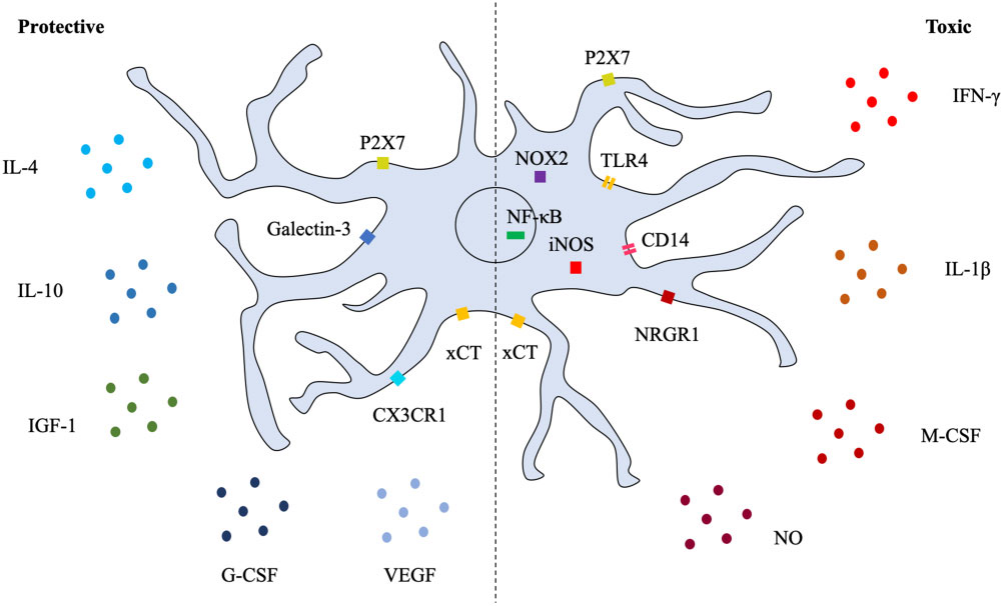
**Several factors have been either directly or indirectly implicated in affecting microglial activation and disease progression in ALS.** Certain receptors including xCT and P2X7 have been linked to both neuroprotection and neurotoxicity depending on the stage of disease progression. Components of this figure were created using Servier Medical Art templates, which are licensed under a Creative Commons Attribution 3.0 Unported License.

Several factors linked to microglial activation have been associated with protection in ALS (Fig. 2). However, only a small number of microglial factors have been directly shown to confer neuroprotection in models of ALS. Several microglia-related cytokines associated with neuroprotection including IL-4, IL-10 and G-CSF have been reported to be altered in ALS patients and models (Jeyachandran *et al.*, 2015; Lu *et al.*, 2016; Moreno-Martinez *et al.*, 2019*a*, *b*). Lentiviral delivery of IL-4 before the onset of disease in mSOD1 mice increased expression of other reportedly neuroprotective genes including *Arg1*, *Retnla* (Fizz1) and *Chil3* (Ym1) and decreased genes associated with neurotoxicity including *Tnfa*, *Ifng* and *Il1b* (Rossi *et al.*, 2018). IL-4 delivery also increased proliferation of microglia while delaying disease onset and improving motor function but did not affect motor neuron loss or lifespan of mSOD1 mice. Administration of IL-10 blocking antibodies increased microglial activation and accelerated onset of motor deficits in mSOD1 mice, whereas microglial-specific lentiviral upregulation of IL-10 delayed disease onset and extended survival of mSOD1 mice (Gravel *et al.*, 2016). However, the effects of IL-10 on motor neuron death in mSOD1 mice were not investigated. Furthermore, G-CSF extended survival in mSOD1 mice, reduced microglial activation and was protective to motor neurons exposed to glutamate excitotoxicity *in vitro*, but did not rescue motor neuron death in mSOD1 mice (Pollari *et al.*, 2011).

Certain growth factors have been directly implicated in microglial neuroprotection. IGF-1 was reported to be decreased in neonatal mSOD1 microglial cultures (Xiao *et al.*, 2007), but increased in later stages of disease in mSOD1 spinal cords (Chiu *et al.*, 2013). Increasing levels of IGF-1 retrovirally reduced microglial activation associated with neurotoxicity, partially rescued motor neuron death, delayed disease progression and extended lifespan of mSOD1 mice (Kaspar *et al.*, 2003; Dodge *et al.*, 2008). Meanwhile, adeno-associated viral delivery of VEGF also partially rescued motor neuron death and motor function, delayed disease progression and was associated with a shift in microglial activation towards a neuroprotective phenotype (Wang *et al.*, 2016). A neuroprotective role for the microglial enriched lectin, galectin-3, has also been proposed as deletion of *Gal3* in mSOD1 mice accelerated disease progression and reduced survival (Lerman *et al.*, 2012). Finally, microglial expression of the fractalkine receptor (CX3CR1) has been associated with neuroprotection in the mSOD1 mouse as mSOD1/CX3CR1 knockout mice displayed increased motor neuron loss and decreased lifespan (Cardona *et al.*, 2006).

### Factors primarily associated with microglial toxicity in ALS

Many factors that are either released from or detected by microglia have been correlated with neurotoxicity in ALS, but as with factors associated with neuroprotection, few have determined direct causality of motor neuron death in models of ALS. Eliminating the expression of one factor may lead to compensatory actions by others, making it difficult to delineate the contribution of individual factors to microglial neurotoxic responses in ALS.

Several cytokines are increased in ALS patients and models (Hensley *et al.*, 2003; Moreno-Martinez *et al.*, 2019*a*). However, few have been shown to directly alter pathology in models of ALS. While TNF-α levels are increased in both ALS patients (Poloni *et al.*, 2000) and symptomatic mSOD1 mice (Hensley *et al.*, 2003), deletion of TNF-α did not affect disease progression or microglial activation in mSOD1 mice (Gowing *et al.*, 2006). Moreover, contradictory evidence has been reported over whether IL-1β affects mSOD1 disease progression. Deletion of *Il1b*extended lifespan and partially rescued motor neuron death of SOD1^G93A^ mice (Meissner *et al.*, 2010), but not SOD1^G37R^ mice (Nguyen *et al.*, 2001). Application of the cytokine M-CSF accelerated disease progression in mSOD1 mice, increasing microglial proliferation and proinflammatory signalling but did not significantly affect motor neuron loss (Gowing *et al.*, 2009). In a recent study, inhibition of IFN-γ with blocking antibodies extended lifespan and improved motor function in mSOD1 mice and shifted microglial gene expression from a neurotoxic to a neuroprotective phenotype (Garofalo *et al.*, 2020).

Upregulation of microglial receptors have also been observed in ALS. Increased expression of toll-like receptors TLR2 and TLR4 and co-receptor CD14 has been reported in post-mortem ALS spinal cord tissue (Henkel *et al.*, 2004; Casula *et al.*, 2011). Transfection of mSOD1 in microglial-like cells has been reported to result in activation of TLR2 (Liu *et al.*, 2009), while deletion of TLR4 improved hindlimb strength and extended lifespan of mSOD1 mice (Lee *et al.*, 2015). Furthermore, activation of proinflammatory signalling resulting from incubation of primary microglia with either mSOD1 (Zhao *et al.*, 2010) or TDP-43 (Zhao *et al.*, 2015) occurred via a CD14-dependent mechanism. The neuroregulin receptor 1 (NRGR1) has been associated with microglial toxicity in ALS as pharmacological inhibition reduced motor neuron death and microglial activation and delayed disease onset and progression in mSOD1 mice (Liu *et al.*, 2018). Increased expression of several purinergic receptors has also been reported in mSOD1 microglia (D'Ambrosi *et al.*, 2009; Cieślak *et al.*, 2019). While P2X7 was implicated in motor neuron death as antagonism of microglial P2X7 rescued SH-SY5Y motor neuron like cells (D'Ambrosi *et al.*, 2009), a more complex role for the P2X7 receptor has been proposed. While knockout of P2X7 accelerated disease in mSOD1 mice (Apolloni *et al.*, 2013), antagonism immediately preceding the onset of symptoms partially ameliorated motor neuron loss and inflammation (Apolloni *et al.*, 2014). Likewise, a dual role for the cystine/glutamate antiporter xCT, which is enriched in microglia, has been proposed as deletion of this receptor in mSOD1 mice initially hastened motor deficits but prolonged the later stages of disease (Mesci *et al.*, 2015).

The transcription factor NF-κB is a master regulator of inflammatory responses that has been linked to microglial involvement in ALS models (Frakes *et al.*, 2014). Several receptor complexes can cause the activation of NF-κB through phosphorylation of the p65 subunit, resulting in the translocation of NF-κB to the nucleus and the upregulation of several proinflammatory factors. Increased phosphorylated p65 has been shown to correlate with disease progression in mSOD1 mice and inhibition of microglial-specific NF-κB extended lifespan of mSOD1 mice (Frakes *et al.*, 2014). Furthermore, inhibition of NF-κB rescued neurotoxicity of co-cultured mSOD1 microglia and reduced the levels of microglial proinflammatory cytokine release (Frakes *et al.*, 2014). The mechanism of mSOD1 activation of NF-κB is not fully understood, but it has been suggested that the microRNA, miR-125b, promotes the activation of NF-κB in microglia by inhibiting the ubiquitin editing enzyme, A20 (Parisi *et al.*, 2016). TDP-43 has also been shown to interact with and activate NF-κB (Swarup *et al.*, 2011*b*). Inhibition of NF-κB was sufficient to improve motor neuron survival in a TDP-43 overexpressing mouse model (Swarup *et al.*, 2011*b*). Furthermore, incubation of microglia with exogenous TDP-43 activated NF-κB and caused motor neuron death in co-culture (Zhao *et al.*, 2015).

Inducible nitric oxide synthase (iNOS) is an enzyme that is upregulated in microglia following activation of NF-κB and is increased in the spinal cord of symptomatic mSOD1 mice (Almer *et al.*, 1999). Inhibition of iNOS was shown to extend lifespan in mSOD1 mice, although the effect on motor neuron survival was not measured (Martin *et al.*, 2007; Chen *et al.*, 2010). iNOS converts the substrate l-arginine to nitric oxide (NO), a molecule that is able to cause motor neuron death *in vitro* (Zhao *et al.*, 2004). Although NO has physiological roles in regulating vasodilation and synaptic transmission, NO can also react with superoxide species to produce the neurotoxic product, peroxynitrite (Calabrese *et al.*, 2007). mSOD1 microglia have been found to produce more NO than wild-type microglia (Beers *et al.*, 2006). NO production in mSOD1 microglia is thought to be NF-κB dependent, as inhibition of NF-κB signalling ablated NO production in mSOD1 microglia (Frakes *et al.*, 2014).

Other sources of reactive oxide species (ROS) have been implicated in microglial-mediated motor neuron death in ALS. NOX2 is a ROS-producing catalytic subunit of the NADPH oxidase that has been implicated in neurotoxicity in ALS. Reduced NOX2 activity has been correlated with increased survival in ALS patients (Marrali *et al.*, 2014) and NOX2 expression was reported to be increased in symptomatic mSOD1 microglia (Chiu *et al.*, 2013). While earlier studies reported that inhibition of NOX2 with apocynin or deletion of *NOX2* partially rescued motor neuron death and extended survival of mSOD1 mice (Wu *et al.*, 2006; Marden *et al.*, 2007; Harraz *et al.*, 2008), these findings were not recapitulated by either genetic or pharmacological means (Seredenina *et al.*, 2016). Together, few factors associated with microglial-mediated neurotoxicity have been directly shown to cause motor neuron death in ALS models. It is likely that a combination of factors act in concert to cause microglial-mediated motor neuron death in ALS.

## Ageing of microglia

Ageing is a strong risk factor for ALS (Pandya and Patani, 2019). Emerging evidence has suggested that the microglial transcriptome undergoes vast changes during normal ageing. Recent studies have reported an increase in inflammatory pathways in microglia during ageing in mice (Clarke *et al.*, 2018; Hammond *et al.*, 2019; Pan *et al.*, 2020). Increased activation of inflammatory pathways has also been observed in human brains across several different regions (Soreq *et al.*, 2017). In this study, changes in gene expression across different regions of the human brain upon ageing were mostly associated with microglia and astrocytes rather than neurons. While mouse and human microglia share many genes, upon ageing human microglia have been shown to undergo more dramatic changes in gene expression (Galatro *et al.*, 2017). Together, these studies show that ageing of microglia has been correlated with an increased inflammatory response, which may partially account for the correlation between ALS and ageing.

It has been reported that microglia increasingly display markers of ageing-associated cellular senescence during disease progression in mSOD1 mice including p16^INK4a^, increased β-galactosidase activity and the loss of nuclear Lamin B1 (Trias *et al.*, 2019). Cellular senescence is an adaptive mechanism that is activated in damaged cells in order to maintain their survival, prevent harmful proliferation and coordinate tissue remodelling (Muñoz-Espín and Serrano, 2014). A direct link between senescence and neurodegeneration has been reported in the MAPT^P301S^PS19 mouse model of Alzheimer’s disease as increased p16^INK4a^ and β-galactosidase activity in microglia and astrocytes was observed in the cortex and hippocampus during disease progression and removal of p16^INK4a^ positive senescent cells reduced neurodegeneration and cognitive decline (Bussian *et al.*, 2018). However, both p16^INK4a^ and β-galactosidase activity have been shown to be reversible in stimulated macrophages and therefore may be distinct from typical cellular senescence pathways (Hall *et al.*, 2017). It remains unknown whether these markers can be used as biomarkers or are actionable drug targets for microglia in ALS.

## Conclusions

The question of whether neuroinflammation is helpful or harmful in ALS is complex and likely determined by a number of factors across the clinical, cellular and molecular levels of the disease. The clinical asynchrony of ALS compounds this complexity, with different cells affected at different times and to different extents. Microglia are unlikely to be the main cell type driving motor neuron death in ALS but may still play an important modulatory role. Both neuroprotective and neurotoxic roles of microglia may co-exist in models of the disease (Liao *et al.*, 2012; Chiu *et al.*, 2013). It is likely that microglia adopt an M2-like neuroprotective state early in the disease but transition to M1-like toxic state as ALS progresses. Given the clinical, cellular and molecular heterogeneity, it is very likely that such transitions in microglial activation state will occur asynchronously.

The key issue of whether activation states of microglia are manipulable for tractable therapeutic benefit has yet to be systematically addressed. Previous trials using general immunosuppression or depleting specific arms of the immune system have not yet led to effective therapies (Crisafulli *et al.*, 2018). Specifically targeting a combination of cytokines either directly or through modulating the activation status of microglia are potentially attractive therapeutic approaches. However, much about the role of microglia in ALS remains unknown. A multimodal approach encompassing *in vitro* and *in vivo* model systems, expressing different ALS causing mutations, will provide further insight concerning the involvement of microglia in ALS. As multiple cell types including astrocytes and peripheral immune cells impact upon microglial function in ALS, determining the mechanisms behind these interactions will provide a more comprehensive understanding of microglia in this disease. Furthermore, understanding the impact of normal ageing on microglia may be important in elucidating how age renders people vulnerable to ALS and other neurodegenerative diseases. Identifying both primary and secondary events in different cell types will help decode the cellular phase in ALS and may lead to polytherapies with a higher success rate in clinical trials.

## Funding

This work was supported by the Francis Crick Institute whichreceives its core funding from Cancer Research UK (FC0101 10), the UK Medical Research Council (FC010110), and the WellcomeTrust (FC010110). R.P. holds an MRC Senior Clinical Fellowship [MR/S006591/1].

## Competing interests

The authors report no competing interests.

## Abbreviations

ALS =: amyotrophic lateral sclerosis
